# NSAIDs in sciatica (NIS): study protocol for an investigator-initiated multicentre, randomized placebo-controlled trial of naproxen in patients with sciatica

**DOI:** 10.1186/s13063-022-06441-3

**Published:** 2022-06-14

**Authors:** Lars Grøvle, Eivind Hasvik, Rene Holst, Anne Julsrud Haugen

**Affiliations:** 1 Department of Rheumatology, Østfold Hospital Trust, Grålum, Norway; 2 Department of Physical Medicine and Rehabilitation, Østfold Hospital Trust, Grålum, Norway; 3 Oslo Centre for Biostatistics and Epidemiology, Faculty of Medicine, University of Oslo, Oslo, Norway; 4 Department of Research, Østfold Hospital Trust, Grålum, Norway

**Keywords:** Nonsteroidal anti-inflammatory drug, NSAID, Naproxen, Sciatica, Low back-related leg pain, Lumbosacral radicular pain, Disc herniation, Randomized controlled trial

## Abstract

**Background:**

Nonsteroidal anti-inflammatory drugs (NSAIDs) are widely used to treat sciatica, despite insufficient evidence from placebo-controlled trials. NSAIDs may cause serious side effects; hence, there is a strong need to clarify their potential beneficial effects in patients with sciatica.

**Methods:**

This is a multicentre, randomized, placebo-controlled, parallel-group superiority trial. Participants will be recruited among sciatica patients referred to outpatient clinics at hospitals in Norway who have radiating pain below the knee with a severity score of ≥ 4 on a 0–10 numeric rating scale and clinical signs of nerve root or spinal nerve involvement. The intervention consists of oral naproxen 500 mg or placebo twice daily for 10 days. Participants will report the outcomes and adverse events daily using an electronic case report form. The primary endpoint is change in leg pain intensity from baseline to day 10 based on daily observations. The secondary outcomes are back pain intensity, disability, sciatica symptom severity, rescue medication (paracetamol) consumption, opioid use, ability to work or study, 30% and 50% improvement in leg pain, and global perceived change of sciatica/back problem. The outcomes will be analysed using mixed effects models for repeated measurements. The total duration of follow-up is 12 (± 2) days.

**Discussion:**

This trial aims to evaluate the benefits of naproxen, a non-selective NSAID, in patients with sciatica. No important differences in efficacy have been demonstrated between different NSAIDs in the management of musculoskeletal disorders; hence, the results of this trial will likely be applicable to other NSAIDs.

**Trial registration:**

ClinicalTrials.gov NCT03347929. Registered on November 20, 2017.

## Administrative information

Note: The numbers in curly brackets in this protocol refer to SPIRIT checklist item numbers. The order of the items has been modified to group similar items (see http://www.equator-network.org/reporting-guidelines/spirit-2013-statement-defining-standard-protocol-items-for-clinical-trials/).

**Table:** 

Title {1}	NSAIDs in sciatica (NIS), an investigator-initiated randomized placebo-controlled trial of naproxen
Trial registration {2a and 2b}	ClinicalTrials.gov NCT03347929 Registered November 20, 2017
Protocol version {3}	Protocol version 2.5, January 21, 2022
Funding {4}	None (internal support provided by Østfold Hospital Trust).
Author details {5a}	Lars Grøvle^1^, Eivind Hasvik^2^, Rene Holst^3,4^, Anne Julsrud Haugen^1^ ^1^Department of Rheumatology, Østfold Hospital Trust, Grålum, Norway ^2^Department of Physical Medicine and Rehabilitation, Østfold Hospital Trust, Grålum, Norway ^3^Oslo Centre for Biostatistics and Epidemiology, Faculty of Medicine, University of Oslo, Norway ^4^Department of Research, Østfold Hospital Trust, Grålum, Norway
Name and contact information for the trial sponsor {5b}	Waleed Ghanima, Department of Research, Østfold Hospital Trust, Pb 300, 1714 Grålum, Norway
Role of sponsor {5c}	Østfold Hospital Trust is a health enterprise owned by the Southern and Eastern Norway Regional Health Authority. Østfold Hospital Trust is responsible for financing, managing, and coordinating the trial.

## Introduction

### Background and rationale {6a}

Sciatica is an established term for pain radiating from the lower back or buttock into the leg. Other commonly used terms are low back-related leg pain, lumbosacral radicular pain, or nerve root pain [1]. The most common source of sciatica is compression and inflammation of the nearby nerve roots and dorsal root ganglia by intervertebral disc herniation [2–4]. In the majority of cases, the L5 and S1 nerve roots are affected, giving rise to pain that radiates below the knee. Patients may also have sensory disturbances, muscular weakness, and low back pain [4]. The estimated prevalence of sciatica is approximately 2–5%, affecting the working-age population [5, 6]. Sciatica may vary from short-lasting, single episodes to a remitting or permanent course over months or years [7, 8]. We previously showed that one-fourth of sciatica patients, who were on sick leave when they were referred to secondary care, were still out of work 2 years later [9].

The treatment of sciatica is primarily aimed at pain reduction by either medication or surgery [1]. The efficacy of analgesic or adjuvant pain medications is uncertain [10]. Paracetamol (acetaminophen) has not been studied in sciatica, and a trial of sustained-release morphine indicated limited effectiveness [11]. Drugs approved for the treatment of painful neuropathy [12, 13], systemic administration of glucocorticoids [14, 15], and biological agents targeting inflammatory cytokines [16, 17] have shown either small or no effects. Epidural corticosteroid injections may offer short-term relief of leg pain and disability, but the effect size is likely small [18, 19].

Given their analgesic and anti-inflammatory mechanisms of action, NSAIDs are widely used to treat sciatica [20]. In a survey of American physicians [21], 80% of respondents said they would recommend NSAIDs for the initial management. A study from general practice in Italy [22] found that 90% of sciatica patients had been prescribed an NSAID. In clinical trials [23, 24], 50–60% of the included patients were taking an NSAID at baseline. However, few placebo-controlled trials investigating the effects of NSAIDs have been performed, and uncertainty exists concerning the potential beneficial effects of NSAIDs in patients with sciatica. The largest study to date investigated the effects of meloxicam 7.5 mg (*n* = 171) and meloxicam 15 mg (*n* = 181) with placebo (*n* = 180) [25]. The difference in overall pain, i.e., back and leg pain, measured on a 100-mm visual analogue scale (VAS), was 5 points lower in both meloxicam groups than in the placebo group at 1 week. Although statistically significant, a 5-point between-group difference is not clinically meaningful [26]. Another trial conducted in Norway between 1988 and 1991 found no differences in leg pain, back pain, or disability between patients taking piroxicam 20 mg (*n* = 120) and placebo (*n* = 94) at 2 weeks [27]. Bontoux et al. [28] reported 9% lower overall pain in patients receiving etodolac (*n* = 62) and 6% lower pain in patients receiving diclofenac (*n* = 59) at 27 h compared to placebo (*n* = 61). Herrmann and Geertsen [29] reported less pain (8–10 mm on a 100-mm VAS) 6 h after the intake of lornoxicam and diclofenac compared with placebo. A Cochrane review of existing trials found that the overall quality of evidence varied from low to very low due to the small study samples, the inconsistent results, and a high risk of bias [30].

Naproxen is a traditional, non-selective NSAID approved for the treatment of inflammatory rheumatic conditions, osteoarthritis, primary dysmenorrhea, and musculoskeletal pain. Similar to other NSAIDs, it provides analgesic, antipyretic, and, in higher doses, anti-inflammatory effects, and it may cause serious gastrointestinal, vascular, and renal side effects [31–33]. There is some evidence that high-dose naproxen is associated with less vascular risk than other NSAIDs [31].

### Objectives {7}

The primary objective is to demonstrate that treatment with naproxen 500 mg twice daily is superior to placebo for the improvement of leg pain intensity in patients with sciatica.

The secondary objective is to demonstrate that naproxen is superior to placebo with respect to the outcomes listed below:

- Improvement in back pain intensity
- Improvement in disability
- The use of paracetamol as rescue medication
- Global perceived improvement
- Improvement in sciatica symptoms
- Thirty per cent and 50% leg pain improvement
- The concomitant use of opioid analgesics
- Improved ability to work and study

### Trial design {8}

This is a multicentre, randomized, placebo-controlled, participant- and assessor-blinded, parallel-group superiority trial.

## Methods: participants, interventions, and outcomes

### Study setting {9}

This trial will take place at outpatient clinics at hospitals in Norway (Østfold Hospital Trust, Vestre Viken Hospital Trust, Telemark Hospital Trust, Stavanger University Hospital, Oslo University Hospital) serving a population of about 1.2 million. Participants will be recruited among sciatica patients referred to the participating centres. To enhance recruitment, primary care physicians will be invited to refer eligible patients.

### Eligibility criteria {10}

The following are the inclusion criteria:

- Age ≥ 18 years
- Radiating pain below the knee with a severity score of ≥ 4 on a 0–10 numeric rating scale (NRS) in the previous 24 h
- Signs of nerve root/spinal nerve involvement as indicated by at least one of the following features: myotomal weakness, dermatomal sensory disturbances (e.g. sensory loss, self-reported tingling/numbness), diminished reflexes, and radiating pain exacerbation by the straight leg raising (SLR) test

The following are the exclusion criteria:

- Not able to read or speak Norwegian
- Unlikely to adhere to treatment and/or complete follow-up (e.g. ongoing serious psychiatric disease, drug abuse, plans to move)
- Sciatica of known cause other than disc herniation or degenerative stenosis
- Neurogenic claudication, i.e. pain in the legs on walking or standing that resolves with sitting down or lumbar flexion
- Symptoms indicating immediate surgery: cauda equina syndrome or progressive large paresis
- Women who are attempting to conceive, are pregnant, or are breastfeeding
- Previous episodes of asthma, urticaria, or allergic-type reactions after taking aspirin or other NSAIDs
- Active or a history of peptic ulceration, gastrointestinal bleeding, or perforation
- Use of drugs known to increase upper gastrointestinal adverse events (AEs) in combination with naproxen: anticoagulants, aspirin (acetyl salicylic acid), serotonin reuptake inhibitors, and systemic corticosteroids
- Hepatic enzyme (ASAT/ALAT) values above 1.5× upper limit of normal (ULN)
- Renal function tests (creatinine/eGFR) outside the normal range
- Congestive heart failure, established ischaemic heart disease, peripheral arterial disease, and/or cerebrovascular disease
- Known hypersensitivity to naproxen or any of the excipients (lactose, maize starch, povidone, sodium starch glycolate, talcum, magnesium stearate, polysorbate 80)
- Ongoing treatment with diuretics, ACE inhibitors, and lithium
- Scheduled for spinal surgery during the study period
- Reservation against the intake of gelatine (the capsules contain gelatine, which among other things is produced with ingredients from pigs)

### Who will take informed consent? {26a}

The investigators (a doctor or a physiotherapist) will obtain written informed consent from the potential trial participants during regular visits to the clinic.

### Additional consent provisions for collection and use of participant data and biological specimens {26b}

N/A. Biological specimens will not be obtained.

### Interventions

#### Explanation for the choice of comparators {6b}

The purpose of this trial is to assess whether naproxen has effects above those of placebo. The comparator in this trial will therefore be an inert placebo. The placebo-controlled trial is widely regarded as the gold standard for testing the efficacy of a treatment. To comply with ethical standards, rescue medication will be permitted.

#### Intervention description {11a}

The intervention consists of 500 mg tablets of naproxen. The comparator consists of placebo tablets containing maize starch and microcrystalline cellulose. Both will be encapsulated using Capsugel® DBcaps®, packaged, and labelled by Kragerø Tablettproduksjon AS, in Kragerø, Norway. The appearance of the capsules, containers, and labelling will be identical for both treatment groups. The study drugs will be administered as 1 tablet twice daily for 10 days, i.e. a total of 20 tablets. The study medication will be dispensed either at the clinics or at the hospital pharmacy.

#### Criteria for discontinuing or modifying allocated interventions {11b}

There will be no modification of the dose or schedule of the study drug.

#### Strategies to improve adherence to interventions {11c}

Participants will be asked to record their intake of the study medication each day via the electronic case report form (eCRF). On day 2, the study staff will check the eCRF; if necessary, they will contact the patient to clear up issues. On day 5, all patients will be contacted by telephone. Adherence will be assessed by pill count. If the pill packages are not returned at the final study visit, adherence will be assessed by the participants’ self-report of study medication consumption.

#### Relevant concomitant care permitted or prohibited during the trial {11d}

A standard package of 100 tablets of paracetamol (acetaminophen) 500 mg will be provided as rescue medication. The dosage will be 1–2 tablets as needed up to a maximum dose of 6 tablets in a 24-h period. Patients will be encouraged to avoid other pain medications. Patients will also be encouraged not to take any other NSAIDs, new anti-depressants, tranquillizers, sleep medications, neuroleptics, or anti-epileptic drugs not on a stable dose prior to the start of the study. The same is true for non-pharmacologic treatments such as physical therapy or acupuncture. Any use of medications or treatments that may affect pain will be recorded and reported but will not be classified as a protocol violation.

#### Provisions for post-trial care {30}

Trial participants will be covered by special insurance for clinical drug trials provided by Norsk Legemiddelforsikring AS, Oslo, Norway. After the final study visit, participants will receive routine medical care. Norway has universal health coverage.

### Outcomes {12}

The primary outcome is the NRS score of leg pain intensity (in the last 24 h). The NRS [34] will be solicited by a presentation of the numbers from 0 to 10, with 0 indicating “no pain” and 10 indicating “pain as bad as you can imagine”. The use of a single pain item is supported by the mission of the Initiative on Methods, Measurement, and Pain Assessment in Clinical Trials [34].

Secondary outcomes are listed below.

- Back pain intensity (in the last 24 h), measured on a 0–10 NRS.
- Disability, assessed by the Roland Morris Disability Questionnaire for Sciatica (RMDQ-S) [35]. The RMDQ-S is a modification of the original RMDQ [36] inquiring explicitly about disability due to back and leg pain.
- Sciatica symptom severity, assessed by the Sciatica Bothersomeness Index (SBI) [37]. The SBI evaluates four sciatica symptoms: (i) leg pain; (ii) numbness or tingling in the leg, foot, or groin; (iii) weakness in the leg/foot; and (iv) back or leg pain while sitting. Each symptom is rated 0–6, and a total score is obtained by summing up the ratings across the four symptoms.
- Rescue medication consumption, measured by pill count, i.e., the number of paracetamol pills not returned at the final study visit. If the paracetamol package is not returned, self-reported data from the electronic diary will be used. Higher rescue consumption indicates more pain.
- Concomitant use of opioids. The use of weak opioids will be quantified using a weighted score by dividing the total dose taken from day 0 to day 10 by its respective defined daily dose (DDD). The DDD is the assumed average maintenance dose per day of a drug used for its main indication in adults. The use of strong opioids will be quantified by the total dose converted into morphine milligramme equivalents.
- Self-reported ability to work or study as usual, assessed by three categories: (i) able, (ii) unable, and (iii) others.
- Thirty per cent and 50% improvement in leg pain.
- Global perceived change (GPC), measured on a verbal rating scale: sciatica/back problem “completely gone”, “much better”, “better”, “a little better”, “no change”, “a little worse”, “worse”, or “much worse”.

The primary endpoint is change in leg pain intensity from baseline to day 10 based on daily observations.

#### Participant timeline {13}

Enrolment and randomization will take place on day 0 (Table 1). No specific washout procedure will be performed, but patients will be encouraged to avoid analgesics and NSAIDs after bedtime on day 0. Study medication will start on day 1 and stop on day 10. On day 2, the study staff will check the eCRF for completeness and contact the patient to clear up issues if necessary. The follow-up on day 5 (± 1) will be conducted by telephone. On day 12 (± 2) or within 7 days after withdrawal, there will be a final study visit at the clinic. Patients will report outcomes in the eCRF at all time points.

**Table 1:**
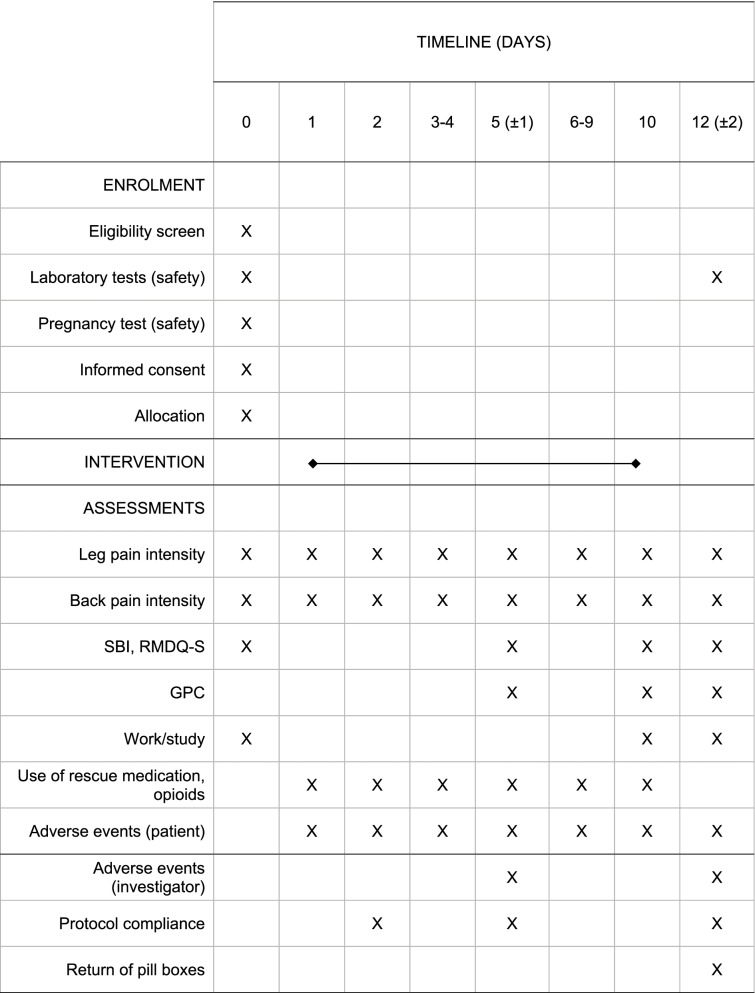
Enrolment and randomization

*SBI* Sciatica Bothersomeness Index, *RMDQ-S* Roland Morris Disability Questionnaire for Sciatica, *GPC* global perceived change

#### Sample size {14}

The sample size estimation is based on a minimum difference of interest between the naproxen group and the placebo group of 1.5 NRS points at day 10. Assuming a standard deviation of 2.5, 90% power and a two-tailed 5% significance level, the inclusion of 60 subjects in each treatment arm is required. The estimated SD is based on data from previous NSAID trials that were included in a Cochrane review [30]. Allowing for a combined dropout and non-compliance rate of ≤ 20%, the total sample size is 150.

### Recruitment {15}

Participants will be recruited among sciatica patients referred to the five participating centres. To enhance recruitment, primary care clinicians will be invited to refer eligible patients.

### Assignment of interventions: allocation

#### Sequence generation {16a}

Subjects will be randomly assigned to receive either naproxen or placebo with a 1:1 allocation as per a computer-generated randomization schedule stratified by site using permuted blocks of random sizes.

#### Concealment mechanism {16b}

The randomization consists of two lists: (i) a central list, generated by a statistician at the CTU, stratified by site and consisting of the site code, randomization number and treatment, and (ii) an allocation list that consists of the drug kit number and treatment. The drug kit numbers will be provided to the approved investigational medicinal product (IMP) manufacturer (Kragerø tablettproduksjon A/S, Kragerø, Norway), who will ensure that the appearance of the containers, the labelling, and the IMP is identical for both treatment groups. The allocation list will be concealed for all other study personnel and participants.

#### Implementation {16c}

Randomization and allocation of the participants will be conducted by the Clinical Trial Unit (CTU), Oslo University Hospital, using Viedoc™ (Uppsala, Sweden), a web-based data capture and management solution. Participants will be enrolled by the study staff at each site. When a patient is allocated to a treatment group using the central randomization list, Viedoc will look for the next available kit numbers for the appropriate treatment group on the allocation list for the subjects’ site and inform the investigator as to which kit should be given to the subject.

### Assignment of interventions: blinding

#### Who will be blinded {17a}

The trial participants, investigators, pharmacists, and data analysts will be blinded to the treatment allocation. At the final study visit, the patients will be asked to guess which treatment they received, with three response options: (i) naproxen, (ii) placebo, or (iii) do not know.

#### Procedure for unblinding if needed {17b}

In the event of a serious adverse event (SAE), unblinding may be performed if the future management of the event necessitates knowledge of the current treatment. The principal investigator (PI) will make this decision. Emergency unblinding can take place at any time using Viedoc. Viedoc will provide a list of all unblindings at the end of the trial.

### Data collection and management

#### Plans for assessment and collection of outcomes {18a}

Investigators and patients will enter data into the Viedoc eCRF. Patients enter data through their smartphone, PC, or tablet using the ViedocMe application. ViedocMe sends reminders at an agreed-upon time. If assessments are not provided, automatic reminders will be sent. Patients who are unable to enter data electronically may use a paper case report form (CRF), and a study collaborator will transfer the data into the eCRF. Except for rescue medication consumption, which will be measured by pill count, all outcome data in this trial are patient-reported (see Outcomes {12} for details).

The eligibility screening includes a clinical examination of the lower extremities (sensory status, muscular strength, reflexes, SLR) and routine laboratory tests (haemoglobin, haematocrit, leucocytes, thrombocytes, creatinine/eGFR, ASAT, ALAT, and ALP). The same laboratory tests will be performed at the final study visit.

Lumbar imaging is not a prerequisite for study participation. Patients who underwent lumbar imaging during the current sciatica episode will have their results recorded as background information. Based on the radiologists’ reports, the results will be categorized as either (i) no changes, (ii) a disc herniation that can explain the symptoms, or (iii) other (specified) changes that can explain the symptoms.

#### Plans to promote participant retention and complete follow-up {18b}

As noted above, participants will receive daily reminders with a link to the eCRF. A paper CRF is provided as a backup in case the eCRF is unavailable. The study staff will check the eCRF on day 2 and day 5; if necessary, they will contact the patient to clear up issues impairing compliance. To reduce unnecessary response burden, the number of questionnaires is limited, and only validated patient-reported outcomes appropriate for sciatica are used. To preserve the intention to treat (ITT) population data will continue to be collected after withdrawal.

#### Data management {19}

A data handling plan was developed by the CTU. Requests to obtain the plan should be addressed to lars.grovle@so-hf.no. Data will be managed using Viedoc, as described above. Database locking will be performed after all data have been entered, and all queries are obtained and solved. After quality control, the database will be specified as SPSS files, exported to the Department of Research at Østfold Hospital Trust, and stored as a comma-separated values (CSV) file for 15 years.

#### Confidentiality {27}

Each site will keep two hard copy lists: (1) a prescreening log of potential participants with initials, eligibility, and reason if not eligible and (2) a list of enrolled participants including name, national identity number, subject ID (generated in Viedoc), and IMP kit number. The lists will be stored in locked cabinets. Access to Viedoc is password-protected.

#### Plans for collection, laboratory evaluation, and storage of biological specimens for genetic or molecular analysis in this trial/future use {33}

Routine laboratory tests will be evaluated according to local reference values. No biological specimens will be collected.

## Statistical methods

### Statistical methods for primary and secondary outcomes {20a}

#### Analysis of the primary outcome

The primary objective of the NIS trial is to estimate the effect of the active treatment relative to the placebo on leg pain intensity from baseline to 10 days after commencement of naproxen or placebo. Multiple measurements taken on the same patient will be correlated. This is accounted for in the analysis by using mixed effects models. The model includes fixed effects for treatment, time (11 time points), the treatment-by-time interaction, and baseline measurements of leg pain plus age and sex. The primary result will be the treatment effect estimates over the period of treatment with 95% CI.

#### Analysis of secondary outcomes

- Back pain intensity will be analysed using the same model used to analyse the primary outcome.
- Disability and sciatica bothersomeness will be analysed using the same model used to analyse the primary outcome, but with 3 time points.
- Responder analyses, i.e. 30% and 50% improvement in leg pain intensity, will be performed using a mixed effects logistic regression model to obtain estimates of odds ratios (OR) and 95% CI. Based on the absolute risk reduction (ARR) between naproxen and placebo, we will calculate the numbers needed to treat (NNT) with 95% CI (NNT = 1/ARR). The ARR and NNT will be presented with 95% CIs.
- Rescue medication consumption and concomitant use of opioids during the treatment period will be analysed using a suitable regression.
- Work/study is an unordered categorical variable. The appropriate model is therefore multinomial with repeated measurements and will be analysed using a GLMM.
- Global perceived change is an ordered categorical variable with repeated measurements and will be analysed using a GLMM.

#### Analysis populations

There will be 3 analysis populations:

1. The intention-to-treat (ITT) population, including all randomized subjects
2. The per-protocol (PP) population, including subjects without any important protocol deviations
3. The safety population, including subjects who received at least one dose of study medication and who had at least one subsequent safety-related observation.

A full statistical analysis plan (SAP) is available on ClinicalTrials.gov.

#### Interim analyses {21b}

The safety profile of naproxen is well-documented and established, and the dosing is under its approved label use. There is no reason to expect naproxen to affect the rate of serious sciatica complications such as lower extremity paresis or cauda equina syndrome. As naproxen will be used under its approved label, no interim safety analyses will be conducted, and stopping guidelines have not been prepared.

### Methods for additional analyses (e.g. subgroup analyses) {20b}

#### Sensitivity analyses

We intend to assess the robustness of the results by:

1. Repeating the primary analysis in the PPP
2. Analyse the primary outcome using multiple imputation (MI)
3. Repeating the primary ITT analysis by including each of the following baseline variables as a covariate
  - Study centre (the stratification variable)
  - Previous NSAID use (yes/no)
  - Imaging findings (disc herniation, yes/no)

We do not plan to analyse subgroups, and no additional analyses are planned to assess the impact of the COVID-19 pandemic on the trial results.

#### Methods in analysis to handle protocol non-adherence and any statistical methods to handle missing data {20c}

The number, timing, pattern, and known reasons for missing values will be assessed and summarized by the treatment group and examined according to baseline characteristics. Missing data will be considered as either missing completely at random (MCAR), missing at random (MAR), or missing not at random (MNAR). If unexpected missing data patterns are found, sensitivity analyses in addition to those mentioned above may be performed.

The LMM and GLMM statistical models for the analysis of the primary outcome and continuous secondary outcomes assume that missing data follow a missing at random (MAR) pattern, in which the probability of missingness may depend on other observed outcome values in the model, but are not related to the unobserved values of missing responses themselves. For outcomes not analysed using likelihood-based methods (LMM and GLMM), missing data will be handled using multiple imputation (MI). MI will also be used in sensitivity analyses.

MI under MAR or MCAR will initially be performed separately within each treatment arm. The models will include all variables in the analytic models plus the values of all baseline characteristics. A total of 50 imputed data sets will be created. Pooled estimates will be calculated using Rubin’s rules.

#### Plans to give access to the full protocol, participant-level data, and statistical code {31c}

The full protocol is available on ClinicalTrials.gov. After study completion, a deidentified and anonymized participant-level dataset will be available upon reasonable request.

### Oversight and monitoring

#### Composition of the coordinating centre and trial steering committee {5d}

The coordinating centre, comprised of the PI and coinvestigators in the Department of Rheumatology, Østfold Hospital Trust, is responsible for the oversight of the trial and will provide day-to-day support. The CTU at Oslo University Hospital will manage the data and monitor the trial. A patient representative from the Norwegian back pain association is a member of the project group.

#### Composition of the data monitoring committee, its role, and reporting structure {21a}

As explained above, this trial involves low risk. The investigational drug naproxen is used under its approved label, and there are no critical safety concerns. No interim safety analyses are planned, and there are no stopping rules. Hence, a data monitoring committee is not needed.

#### Adverse event reporting and harms {22}

This trial complies with the Norwegian national research infrastructure body (NorCRIN) standard operating procedure for safety reporting in clinical drug trials [38]. Patients will be asked to report potential AEs daily. The eCRF includes a check-off list of 10 expected non-serious AEs and an open text field. Additionally, investigators will inquire about AEs at the follow-up on day 5 and at the final study visit. Serious adverse events (SAEs) and suspected unexpected serious adverse reactions (SUSARs) will be handled and reported according to the standard operating procedure.

#### Frequency and plans for auditing trial conduct {23}

A monitor at the CTU, independent from the sponsor and investigators, will monitor the trial according to a comprehensive monitoring plan. The monitor will verify the compliance to study protocol and procedures, check the labelling and handling of the study drugs, and the registration of AEs. On-site monitoring visits will be performed at initiation, during the study (once per year), and at close-out. There will be no trial audit but authorized representatives of the Norwegian Medicines Agency and the Norwegian Regional Ethics Committee South East may perform inspections, including source data verification.

#### Plans for communicating important protocol amendments to relevant parties (e.g., trial participants, ethical committees) {25}

Important protocol modifications will be distributed to the investigators, the Norwegian Medicines Agency, the Norwegian Regional Ethics Committee South East, and ClinicalTrials.gov.

#### Dissemination plans {31a}

The results will be submitted for publication in a peer-reviewed journal, presented at scientific conferences and uploaded to ClinicalTrials.gov. We will also communicate the results to the Norwegian Back Pain Association, the press, and social media.

## Discussion

This trial seeks to clarify the effects of a non-selective NSAID, namely, naproxen, in patients with sciatica. No important differences in efficacy have been demonstrated between NSAIDs in the management of musculoskeletal disorders [39]; hence, the results of this trial will likely be applicable to other NSAIDs. Our choice of naproxen was based on reports indicating less vascular risk than that associated with other NSAIDs [31].

Initial decisions in sciatica treatment are usually based on patient history and clinical examination. To reflect clinical practice, lumbar imaging is not a prerequisite for study enrolment. Occasionally, it may be difficult to distinguish between radicular and referred pain, but in the latter, neurological signs are generally absent. Our requirement of pain radiating below the knee and at least one neurological sign should ensure that a nerve root is affected. For patients referred to secondary care, lumbar MRI or CT images are usually available at the time of consultation, and the results will be known to investigators prior to enrolment. Although unlikely, we cannot rule out that trial candidates with distinct imaging findings may be more prone to be enrolled than candidates with uncertain findings. Furthermore, candidates’ previous experiences with NSAIDs may affect their willingness to participate and thereby affect the generalizability of the results of this trial. Candidates who experienced side effects or felt that treatment was not helpful may be less willing to participate than candidates with positive experiences. The widespread use of NSAIDs to treat pain, fever, sprains, arthritis, etc. makes it unrealistic to include NSAID-naïve patients only. To test the robustness of the trial results, imaging findings and previous NSAID use will be included in the sensitivity analyses.

We consider a 10-day treatment period to be sufficient to assess the effect of naproxen on leg pain. Peak plasma levels are reached 2 to 4 h after oral administration, and a steady state is achieved within 3 days of initiation of therapy on a twice-daily dose regimen [40]. When NSAIDs are taken for 10 days or fewer, most patients are not at an increased risk of developing serious AEs [41]. Evidence for an effect in this trial would justify a longer-term trial.

Sciatica patients commonly report a combination of leg pain, back pain, neurological disturbances, and disability. To determine the full effects of naproxen, these aspects will be covered. Global variables include the RMDQ-S, the SBI, and the GPC. To reduce within-subject variability and increase study power, the outcomes will be analysed using mixed effects models for repeated measures.

There is no robust evidence for the clinically important between-group difference in leg-pain intensity in sciatica. Generally, the difference in the magnitude of response between the treatment and control groups that will be considered large enough to establish the therapeutic importance of the results should be established in the context of the disease being treated, the available treatments, and the risk-benefit ratio of the treatment [26, 42]. In this present trial, the target difference was set to 1.5 on the 0–10 NRS. Previous trials have used 1.5 [13] and 1.0 [43]. We acknowledge that smaller differences than 1.5 could be relevant. Due to the lack of data required for estimating sample size for mixed effects models, such as the correlations among the daily measurements, the sample size for this trial was calculated using *t*-test [44]. A mixed effects model would likely have resulted in a lower sample size estimate [45]. To enhance the interpretability of the results, we will also perform responder analyses, i.e. the number of participants who achieve 30%, and 50%, improvement in leg pain. These thresholds are considered to represent moderate and substantial clinically important change, respectively [42, 46].

## Trial status

The current protocol is version 2.5, 21 January 2022. The first participant was randomized in December 2017; as of April 2022, 103 subjects have been randomized (Østfold Hospital Trust (*n* = 80), Vestre Viken Hospital Trust (*n* = 0), Telemark Hospital Trust (*n* = 20), Stavanger University Hospital (*n* = 1), Oslo University Hospital (*n* = 2)). The Vestre Viken Hospital site was closed in 2021. Recruitment was not paused during the COVID-19 pandemic. Recruitment is expected to be completed by the first half of 2023.

## Data Availability

The investigators will have full access to the final dataset. The sponsor will not have access to the data or be involved in the analysis, interpretation or writing of the study results, or decision to submit the report for publication.

## Abbreviations

AE: Adverse event
ARR: Absolute risk reduction
ALAT: Alanine transaminase
ASAT: Aspartate aminotransferase
ALP: Alkaline phosphatase
CRF: Case report form
CSV: Comma-separated values
CTU: Clinical trial unit
DDD: Defined daily dose
eCRF: Electronic case report form
GPC: Global perceived change
GLMM: Generalized linear mixed model
IMP: Investigational medicinal product
LMM: Linear mixed model
NNT: Number needed to treat
NRS: Numeric Rating Scale
NSAID: Nonsteroidal anti-inflammatory drug
PI: Principal investigator
RMDQ-S: Roland Morris Disability Questionnaire for Sciatica
SAE: Serious adverse event
SAP: Statistical analysis plan
SUSAR: Suspected unexpected serious adverse reaction
SBI: Sciatica Bothersomeness Index
SLR: Straight leg raising
ULN: Upper limit of normal
